# AI-Enhanced Healthcare: Integrating ChatGPT-4 in ePROs for Improved Oncology Care and Decision-Making: A Pilot Evaluation

**DOI:** 10.3390/curroncol32010007

**Published:** 2024-12-26

**Authors:** Chihying Liao, Chinnan Chu, Mingyu Lien, Yaochung Wu, Tihao Wang

**Affiliations:** 1 Department of Radiation Oncology, China Medical University Hospital, Taichung City 404, Taiwan; 014069@tool.caaumed.org.tw (C.L.); 014360@tool.caaumed.org.tw (C.C.); 2 Graduate Institute of Clinical Medical Science, China Medical University, Taichung City 404, Taiwan; 3 Division of Hematology and Oncology, Department of Internal Medicine, China Medical University Hospital, Taichung City 404, Taiwan; 012604@tool.caaumed.org.tw; 4 Department of Surgery, China Medical University Hospital, Taichung City 404, Taiwan; 028097@tool.caaumed.org.tw; 5 Department of Medicine, China Medical University, Taichung City 404, Taiwan

**Keywords:** cancer, side effect, ePRO, ChatGPT-4, healthcare, AI

## Abstract

Background: Since 2023, ChatGPT-4 has been impactful across several sectors including healthcare, where it aids in medical information analysis and education. Electronic patient-reported outcomes (ePROs) play a crucial role in monitoring cancer patients’ post-treatment symptoms, enabling early interventions. However, managing the voluminous ePRO data presents significant challenges. This study assesses the feasibility of utilizing ChatGPT-4 for analyzing side effect data from ePROs. Methods: Thirty cancer patients were consecutively collected via a web-based ePRO platform, reporting side effects over 4 weeks. ChatGPT-4, simulating oncologists, dietitians, and nurses, analyzed this data and offered improvement suggestions, which were then reviewed by professionals in those fields. Results: Two oncologists, two dieticians, and two nurses evaluated the AI’s performance across roles with 540 reviews. ChatGPT-4 excelled in data accuracy and completeness and was noted for its empathy and support, enhancing communication and reducing caregiver stress. It was potentially effective as a dietician. Discussion: This study offers preliminary insights into the feasibility of integrating AI tools like ChatGPT-4 into ePRO cancer care, highlighting its potential to reduce healthcare provider workload. Key directions for future research include enhancing AI’s capabilities in cancer care knowledge validation, emotional support, improving doctor-patient communication, increasing patient health literacy, and minimizing errors in AI-driven clinical processes. As technology advances, AI holds promise for playing a more significant role in ePRO cancer care and supporting shared decision-making between clinicians and patients.

## 1. Introduction

ChatGPT, an advanced AI Large Language Model (LLM) technology, was launched by the American artificial intelligence research lab OpenAI at the end of November 2022 [1]. Through extensive data training, ChatGPT assists in analyzing medical records [2], providing psychological health support [3], and aiding medical research in the field of medicine [4]. This technology is reshaping our medical practices, sparking a new revolution in healthcare [5]. In March 2023, OpenAI released ChatGPT-4, a more human-like general AI than its predecessor, ChatGPT-3.5 [1]. It demonstrates superior performance in understanding, reasoning, and responding to complex questions. By September 2023, OpenAI updated ChatGPT to perform internet searches through Microsoft’s Bing search engine, breaking free from the data limitations of September 2021 [6].

In the medical field, ChatGPT-4, through its deep learning and natural language processing capabilities, has passed the three-stage United States Medical Licensing Examination (USMLE) [7]. This marks a significant milestone in the maturity of AI in healthcare. It can assist medical professionals in handling a vast amount of medical literature [8], clinical records, insurance documents, and patient inquiries [9]. ChatGPT-4 can provide information on clinical trials relevant to patient needs [2], assist doctors in quickly accessing information on related cases, and offer suggestions based on the latest medical education and research [10], thereby enhancing work efficiency and the accuracy of information processing.

In the field of cancer, ChatGPT-4 can assist in patient education for cancer patients, analysis of next-generation genetic data in cancer (NGS) [2], care recommendations for patients with hepatitis and cirrhosis [11], clarifying common cancer myths [12,13], and answering health-related questions on social media [3]. While ChatGPT-4 provides professional and accurate responses and its conversational manner is warm, empathetic [3], and patient, it is not yet able to provide complete and correct guidance for cancer treatment [13,14].

In modern cancer care, the increasing application of electronic patient-reported outcomes (ePROs) underscores their importance in enhancing patient care quality and treatment effectiveness [15,16]. EPROs allow patients to report their health status, symptoms, and quality of life through digital platforms, providing valuable firsthand data for medical teams [17]. This approach not only helps physicians understand and manage symptoms more precisely but also gives patients a greater sense of participation and control during treatment [18].

Increasing research shows that the care model of electronic patient-reported outcomes (ePROs) provides patient data that can be increasingly integrated into clinical decision support tools [18,19,20,21,22]. This aids healthcare professionals in timely identifying and addressing potential health issues, thereby preventing disease progression or mitigating side effects. Recent ePROs research can help patients improve treatment tolerance, improve survival rates, enhance communication and interaction between doctors and patients, reduce unnecessary emergency department visits and hospitalizations [16], and decrease medical expenses, leading to better cost-effectiveness [23]. Additionally, the accumulation of ePROs data offers a rich resource for clinical research, contributing to improved future cancer treatment strategies and patient care models [24]. It also helps reduce carbon emissions, aiding hospital ESG (environmental, social, and governance) transformation [23,25], and provides better cancer care during the COVID-19 pandemic [15].

However, ePROs generate vast amounts of electronic symptom data from patients, necessitating additional nurses and time for interpretation, analysis, and further generation of clinical decision-making processes for symptom management [19,21]. For instance, when a patient reports persistent diarrhea accompanied by significant weight loss, healthcare providers may analyze the data to determine whether immediate intervention, such as a telephone reminder to return to the hospital for examination, is necessary. Additionally, this process may require collaboration with a dietitian to provide tailored health education on nutrition and hydration strategies, aiming to mitigate further deterioration and improve the patient’s quality of life.

In a medical environment marked by a shortage of oncology staff and overburdened healthcare personnel [26,27], ePROs may contribute to an increased workload. However, integrating ChatGPT-4 to analyze medical data, drive innovation, assist with patient discharge notes, and provide educational support could help alleviate the burden on healthcare teams and enhance patient care [2,4,9,10,28].

We conducted a pilot study utilizing ChatGPT-4 to analyze ePRO data on patient-reported side effects. ChatGPT-4 was employed to simulate various roles within the medical team, aiming to analyze side effect data across different cancer types, patient age groups, and treatment modalities, while providing recommendations for improvement. The medical team subsequently evaluated the feasibility of incorporating ChatGPT-4’s analytical responses into clinical decision-making processes.

## 2. Materials and Methods

### 2.1. Data Collection and Preparation

We utilize a web-based electronic patient-reported outcome system (developed by Cancell Tech Co., Ltd., Taipei , Taiwan, available at [29]), which was launched in January 2023 for use by cancer patients. Each patient can create their own account with a password, agree to the terms of use for clinical study by signing a consent form, and then log in to use the system. Patients are required to fill in basic information such as name, gender, age, type of cancer, stage, and current treatment. During their treatment, patients subjectively report symptoms of side effects twice a week using the electronic patient-reported outcome system, which incorporates common cancer side effects as defined by NCI/ASCO [16,30]. The data recorded by the patients include quantifiable metrics such as weight and body temperature; scores for quality of life and mood on a scale from 1 to 5, where higher scores indicate better states; and pain scores also ranging from 1 to 5, with higher scores indicating more severe pain. Symptoms such as reduced appetite, stomach discomfort, diarrhea, constipation, nausea and vomiting, coughing, shortness of breath, fatigue, depression, and insomnia are rated on a four-point scale: none for no symptoms; mild, moderate, and severe symptoms; and radiation dermatitis using grading, covering a total of 16 symptom items. The data are stored in AWS cloud storage, with firewalls and other cybersecurity measures in place for data protection.

To gain an initial understanding of the ePRO data analysis for different cancer types, we consecutively collected 30 patients who had completed the ePRO side effect data for at least 4 weeks from September 2023 to December 2023. We de-linked and de-identified the basic information of these patients, retaining only their age, type of cancer, gender, stage, and treatment method, along with the ePRO side effect symptom data, for further analysis by ChatGPT-4.

**Figure 1:**
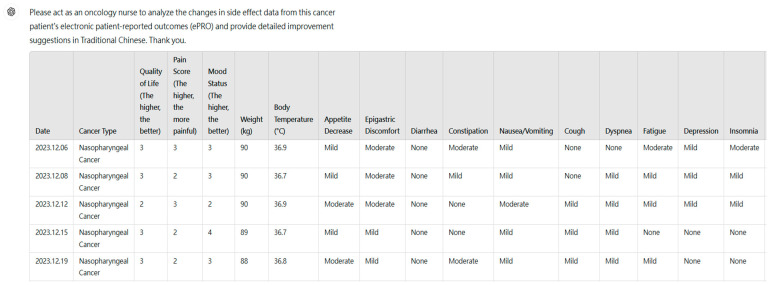
Example of ePRO data from a nasopharyngeal cancer patient used to prompt GPT-4 to act as a nurse for evaluating the data.

Among the 30 de-identified patients, an example of ePRO side effect data collected during treatment for nasopharyngeal cancer is presented. The data was formatted in Excel and then copied and pasted into a new conversation in ChatGPT-4 on the OpenAI website [31], as shown in Figure 1.

### 2.2. ChatGPT-4 Prompt

We referred to the prompt engineering recommendations from the OpenAI website (https://platform.openai.com/docs/guides/prompt-engineering (accessed on 5 January 2024) [32] and adopted the “adopt persona” approach to have ChatGPT-4 simulate three types of professionals. For each new conversation, we made a prompt to request ChatGPT-4 to simulate the roles of an oncologist, a dietitian, and a nurse, respectively, to analyze the changes in 4 weeks of ePRO side effect data for these 30 patients undergoing cancer treatment, providing analysis and improvement suggestions, as shown in Figure 1. The prompt used was as follows: “Please act as an oncologist (or a dietitian, or a nurse) to analyze the changes in the side effect data from this cancer patient’s electronic patient-reported outcomes (ePRO) and provide detailed improvement suggestions in Traditional Chinese. Thank you.” It was the same for all patients. We made 90 prompts and then collected 90 corresponding responses across the three expert roles of 30 patients generated by ChatGPT-4. (Each patient received 3 sets of expert simulations by ChatGPT-4.) An example of a response from GPT-4 acting as a nurse for a nasopharyngeal cancer patient’s ePRO data is shown in Table 1.

### 2.3. Evaluation Workflow

Thirty patients received a total of 90 sets of recommendations generated by ChatGPT-4 across the three expert roles.

**Figure 2:**
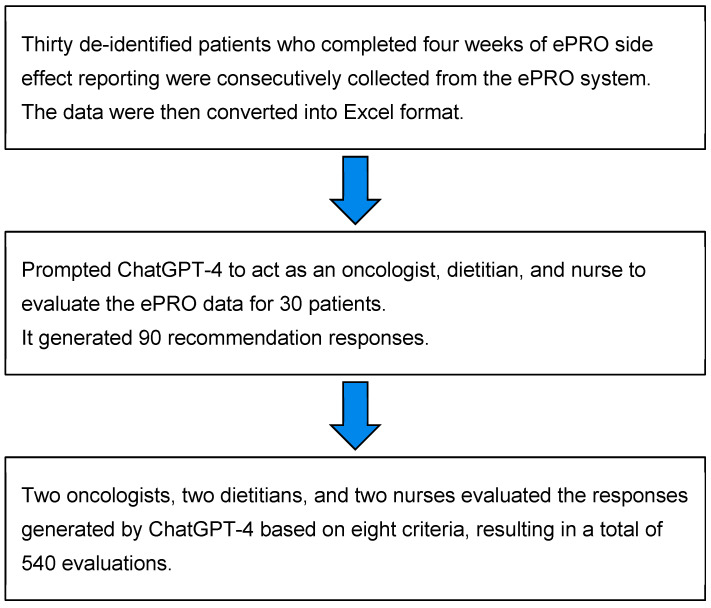
The evaluation workflow of this study.

These recommendations were then evaluated by a senior medical team comprising 2 oncologists, 2 oncology dietitians, and 2 oncology nurses, resulting in 180 evaluations per role, totaling 540 evaluations. The medical teams evaluated all responses, including the patient’s ePRO data and ChatGPT-4’s replies, using eight criteria grounded in their professional expertise. The eight criteria were: response completeness, content accuracy, minimized risk to the patient, empathy demonstrated in the reply, emotional support provided, improvement in patient communication efficiency, the potential of ChatGPT-4 to alleviate the medical care workload when used cautiously, and its effectiveness in enhancing patient health literacy regarding their disease. The evaluations were scored on a scale from 0 to 10, with 0 representing very poor and 10 representing very good evaluation, with examples in Table 1. ANOVA statistical analysis was used to determine if there were any significant differences among the eight evaluation criteria for the three simulated professions by ChatGPT-4. This study workflow is shown in Figure 2.

The study was approved by the institutional review board of China Medical University and Hospital, Taichung, Taiwan (No.:ePRO_HN_001/CMUH112-REC2-128).

## 3. Results

Among the 30 consecutively selected cancer patients, there were eight cases of breast cancer, seven cases of head and neck cancer, three cases of lung cancer, and two cases each of prostate cancer, pancreatic cancer, lymphoma, renal cell carcinoma, uterine sarcoma, and endometrial cancer. In terms of cancer stages, there were five cases of stage I, seven cases of stage II, eight cases of stage III, and ten cases of stage IV. The median age was 55 years. Regarding treatment methods, 12 patients received chemotherapy, 8 underwent concurrent chemoradiotherapy, 5 had radiotherapy, and 5 were given targeted therapy. There were 11 male patients and 19 female patients. The patients’ characteristics are in Table 2.

The two oncologists evaluating GPT-4 included a radiation oncologist and a hematologist-oncologist. Furthermore, two oncologists, two oncology nutritionists, and two oncology nurses assessed GPT-4’s simulated performance across three roles in the treatment of 30 patients, resulting in 180 evaluations per role, totaling 540 evaluations.

When ChatGPT-4 performed as an oncologist, dietitian, and nurse in analyzing the ePRO data of 30 patients, the average evaluation scores for each role are presented in Table 3.

ChatGPT-4 was assigned three roles: oncologist, dietitian, and nurse. Analysis of ePRO data from 30 patients across eight criteria, using ANOVA, revealed no significant differences (*p* > 0.05) among the three roles, as shown in Table 3. The average scores across the eight criteria were 6.85/10 for the oncologist, 7.31/10 for the dietitian, and 7.16/10 for the nurse. ChatGPT-4 achieved its highest performance in the role of dietitian. There are some examples of good responses from ChatGPT-4 in Table 4**.**

## 4. Discussion

In this study, electronic patient-reported outcomes (ePROs) were employed to gather data on patients’ self-reported health status, symptoms, and quality of life via digital devices. While the extensive data from ePROs facilitates early intervention and mitigates side effects, it also significantly increases the workload for medical teams [19,20]. ChatGPT-4 demonstrated its potential in effectively processing and analyzing these vast volumes of data, enabling medical professionals to better comprehend patient needs and respond in an empathetic and emotionally supportive manner [33].

The analysis of ePRO data in traditional Chinese from 30 consecutive cancer patients by ChatGPT-4 yielded ratings from “acceptable” to “good”; the average score achieved was 6.85 to 7.31, showing no significant differences when simulating the roles of oncologists, dietitians, and nurses. ChatGPT-4 particularly excelled in completeness and accuracy, indicating its effectiveness in processing ePRO data and identifying critical side effects. This underscores the potential of AI to enhance clinical decision-making and patient care, particularly by aiding healthcare professionals in managing extensive patient-reported outcomes amidst a demanding medical workflow.

The minimal patient harm observed can likely be attributed to the careful limitation of ChatGPT-4’s evaluation to ePRO content only [34]. This suggests a need for continued caution when employing AI technologies based on large language models. Future improvements in AI response accuracy and correctness may be achieved through retrieval-augmented generation (RAG), which could help prevent incorrect answers that might negatively impact patients [35]. Nonetheless, the moderate scores for empathy and emotional support highlight AI’s limitations in fully addressing the emotional needs of patients, suggesting that AI tools should be used in conjunction with personal interactions from healthcare professionals to meet comprehensive patient needs comprehensively [33].

Moreover, this study initially demonstrates AI’s potential to assist medical teams in utilizing ePROs for enhanced patient care, improving communication efficiency with patients, reducing medical care stress, and increasing health literacy. These results support further research to explore and integrate ChatGPT-4 and other AI technologies into clinical ePRO applications for cancer care, aiming to foster patient-centered care and improve treatment outcomes.

While the recommendations provided by ChatGPT-4 in the roles of oncologist, dietitian, and nurse were statistically similar, the model received higher evaluations when simulating the role of an oncology dietitian. This may reflect the significant role of nutritional education in managing cancer side effects via ePROs and highlights the importance of nutritional support in enhancing patient quality of life. It could also suggest that ChatGPT-4 has specific advantages in delivering nutritional literacy and education, emphasizing the unique and crucial role of a virtual dietitian in patient care [28].

This pilot study encountered several limitations, such as a small sample size and issues with the misidentification of worsened ePRO side effects. The diversity of the data input into ChatGPT, including a range of cancer types, stages, treatments, and patient demographics, added complexity but also presented unique opportunities. This heterogeneity allowed us to test the model’s ability to generalize across varied clinical scenarios, which is crucial for its applicability in real-world settings. However, it also posed challenges in ensuring the consistency and accuracy of the AI’s outputs, as well as in verifying the authenticity of patient-reported ePRO data. Potential bias may exist since ChatGPT’s performance was evaluated by only six professionals. Additionally, as a large language model, ChatGPT-4 was not specifically designed to adhere to the Health Insurance Portability and Accountability Act (HIPAA) [2], posing potential risks associated with race, age, and gender disparities. Furthermore, the model’s lack of accuracy and verified sources in providing cancer treatment recommendations warrants a cautious approach [13,14].

A further limitation is the degree to which ChatGPT-4 truly understands the distinctions among the roles of oncologist, dietitian, and nurse. The prompt to “act like an [expert]” remains somewhat open-ended, and it is unclear how ChatGPT-4 interprets each professional domain. This ambiguity may influence the validity of role-based responses and warrants more detailed instructions or context to ensure accurate role simulation.

In practical applications, the use of large language models like ChatGPT-4 in healthcare must consider data privacy, model bias, and the safe use of AI in medical decision-making processes. Future developments should balance technological innovation with legal and medical ethics to ensure that AI not only enhances healthcare efficiency but also safeguards patient rights and interests [10].

Overall, this study provides preliminary insights into the feasibility of integrating AI tools like ChatGPT-4 into ePRO cancer care. It highlights the potential to reduce healthcare provider workload and suggests directions for future research and applications. These include enhancing AI’s capability to revalidate cancer care knowledge through techniques like RAG and multimodal LLMs. Additionally, AI can provide emotional support and empathy, enhance doctor-patient communication, increase patient health literacy, reduce the burden on healthcare providers, minimize potential errors in AI-driven clinical processes, and further explore the optimal application of AI in various medical roles. With continued technological advancements and empirical research, AI has the potential to play a more significant role in ePRO cancer care and facilitate shared decision-making between clinicians and patients.

## Figures and Tables

**Table 1:** Example of a response of GPT-4 acting as a nurse to evaluate a nasopharyngeal cancer patient’s ePRO data, with subsequent review by the oncologist, dietitian, and nurse.

Patient	ChatGPT-4 Responses of Acting as Nurse	Example of the Expertise Evaluations
Nasopharyngeal cancer patient’s ePRO data.	**Analysis of ePRO Side Effect Data** **1.** **Quality of Life and Mood Status** **Quality of Life (QoL)**: The patient’s QoL fluctuates throughout the month, with a notable decline to **2** on 12 December 2023, 22 December 2023, and 30 December 2023. This may reflect a cumulative impact of symptoms like fatigue, nausea/vomiting, and epigastric discomfort. **Mood Status**: Mood follows a similar trend, with dips on 12 December 2023, 22 December 2023, and 30 December 2023. This may indicate emotional distress associated with worsening symptoms. **2.** **Pain Management** The pain score peaked at **3** on 6 December 2023 and 12 December 2023, coinciding with moderate levels of epigastric discomfort, nausea, and fatigue. Pain management strategies appear effective between 8 December 2023 and 30 December 2023, with scores stabilizing at **2**. **3.** **Appetite and Weight Trends** **Appetite**: The patient’s appetite decreased to “Moderate” on multiple dates, likely contributing to a gradual weight loss from **90 kg** on 6 December 2023 to **87 kg** on 30 December 2023. Addressing underlying symptoms (e.g., epigastric discomfort and nausea) is crucial for maintaining nutritional status. **4.** **Fatigue and Insomnia** Fatigue is consistently reported as “Mild” to “Moderate”, with no days marked as “None”. Persistent fatigue, coupled with moderate levels of insomnia (e.g., 6 December 2023), may further impact QoL and mood. **5.** **Radiation Dermatitis** Radiation dermatitis emerges as a **Grade 1** issue starting on 19 December 2023. While mild, it warrants monitoring to prevent progression.	1 oncologist Completeness: 8 Accuracy: 8 Minimal Risk to the patient: 9 Empathetic response: 6 Emotional support: 6 Improvement in patient communication: 8 Reduction in medical care stress: 8 Increased in health literacy: 7 1 dietitian Completeness: 8 Accuracy: 8 Minimal Risk to the patient: 9 Empathetic response: 7 Emotional support: 6 Improvement in patient communication: 7 Reduction in medical care stress: 8 Increased in health literacy: 8 1 nurse Completeness: 9 Accuracy: 9 Minimized risk to the patient: 9 Empathetic response: 7 Emotional support: 6 Improvement in patient communication: 8 Reduction in medical care stress: 8 Increased in health literacy: 8
	**Improvement Suggestions** **1.** **Symptom-Specific Management** **Pain**: Ensure optimal pain control by reviewing the current analgesic regimen. Consider non-pharmacological options, such as relaxation techniques, if appropriate. **Fatigue**: Encourage light physical activity (e.g., short walks) and prioritize energy-conserving strategies to mitigate fatigue. **2.** **Nutritional Support** Collaborate with a dietitian to provide high-calorie, nutrient-dense meals and snacks to address appetite loss and stabilize weight. Soft or easy-to-digest foods may help reduce epigastric discomfort. **3.** **Radiation Dermatitis Care** Educate the patient on proper skin care, including moisturizing and avoiding irritants. If symptoms worsen, consider low-dose topical corticosteroids or other medical interventions. **4.** **Mood and Sleep Interventions** Refer the patient for counseling or psychosocial support to address mood dips and depression. Sleep hygiene education (e.g., regular sleep schedule, limiting screen time before bed) may help alleviate insomnia. **5.** **Regular Monitoring** Schedule regular follow-ups to monitor the progression of side effects, especially fatigue, radiation dermatitis, and appetite-related concerns, to adapt treatment plans promptly. By addressing these areas, the patient’s overall quality of life and symptom management during treatment can be significantly improved.	

**Table 2:** Patient characteristics.

Characteristics	Numbers
Breast cancer	8
Head neck cancer	7
Lung cancer	3
Prostate cancer	2
Pancreas cancer	2
Lymphoma	2
Renal cell carcinoma	2
Uterine sarcoma	2
Endometrial cancer	2
Stage	
I	5
II	7
III	8
IV	10
Treatment modality	
Chemotherapy	12
Concurrent chemoradiotherapy	8
Radiotherapy	5
Target therapy	5

**Table 3:** Scores of GPT-4 acting as an oncologist, dietitian, and nurse evaluating ePRO data and ANOVA statistical analysis.

	Act as Oncologist	Act as Dietitian	Act as Nurse	*p*-Value
Completeness	7.3 (4–10)	7.2 (6–10)	7.3 (5–9)	*p* = 0.881
Accuracy	7.6 (3–9)	7.7 (4–9)	7.7 (0–9)	*p* = 0.149
Minimal risk	9.4 (7–10)	9.2 (7–10)	9.5 (7–10)	*p* = 0.507
Empathetic response	5.0 (0–9)	6.0 (4–9)	5.4 (1–10)	*p* = 0.187
Emotional support	4.8 (0–9)	5.9 (2–9)	5.3 (0–10)	*p* = 0.101
Improvement in patient communication efficiency	6.7 (1–9)	7.3 (2–9)	7.0 (1–9)	*p* = 0.242
Reduction in medical care stress	7.2 (1–10)	7.6 (2–10)	7.8 (1–10)	*p* = 0.197
Increase in health literacy	6.9 (2–10)	7.6 (2–10)	7.3 (3–10)	*p* = 0.08
Average score	6.85	7.31	7.16	

**Table 4:** The examples of good responses from ChatGPT-4.

ChatGPT-4.0 Role Simulation	ChatGPT-4 Evaluations and Responses
Oncologist	**Evaluation** Radiation Dermatitis: Emerges on 12/19 as Grade 1 and persists but does not progress to a higher grade. Grade 1 dermatitis usually involves mild erythema or dryness. **Response** Skin Hygiene: Cleanse with lukewarm water and mild soap, gently pat dry. Apply physician-recommended topical agents (e.g., moisturizing lotions, low-potency steroids) if needed. Skin Protection: Avoid tight clothing or harsh chemicals. Monitor daily for changes such as increased redness or peeling.
Dietitian	**Evaluation** Weight Changes: Decrease from 90 kg to 87 kg suggests progressive weight loss, potentially due to moderate appetite decrease and persistent GI issues (e.g., constipation). Ongoing malnutrition risk warrants nutritional intervention. **Response** Nutritional Support: Calorie-Dense Diet: Incorporate high-calorie, high-protein options (e.g., lean meats, dairy, beans) to address weight loss and support healing. Small Frequent Meals: Help the patient manage appetite decrease by dividing meals into smaller portions throughout the day. This strategy can also ease epigastric discomfort. Nutritional Supplements: If food intake remains insufficient, consider oral supplements or consult a dietitian for a tailored meal plan.
Nurse	**Evaluation** Fatigue: Fatigue is consistently reported as “Mild” to “Moderate”, with no days marked as “None”. Persistent fatigue, coupled with moderate levels of insomnia (e.g., 6 December 2023), may further impact QoL and mood. **Response** Fatigue: Encourage light physical activity (e.g., short walks) and prioritize energy-conserving strategies to mitigate fatigue. Refer the patient for counseling or psychosocial support to address mood dips and depression. Sleep hygiene education (e.g., regular sleep schedule, limiting screen time before bed) may help alleviate insomnia.

## Data Availability

The study detail data are unavailable due to privacy or ethical restrictions.
